# Supplementary material for: Development of a computer-aided design software for the quantitative evaluation of aesthetic damage
Source: PLoS One. 2019 Dec 18;14(12):e0226322. doi: 10.1371/journal.pone.0226322 (PMC6919621; doi:10.1371/journal.pone.0226322)
Supplement: S1 Code site — 1. (ZIP) [file pone.0226322.s001.zip › [EESC_jr][Projeto]Relatorio_07_21/vendors/select2/docs/_includes/options/selections/clearing-selections.html]

## Can I allow users to clear their selections?

You can allow people to clear their current selections with the `allowClear` option when initializing Select2. Setting this option to `true` will enable an "x" icon that will reset the selection to the placeholder.

{% highlight js linenos %}
$('select').select2({
placeholder: 'This is my placeholder',
allowClear: true
});
{% endhighlight %}

### Why is a placeholder required?

{% include options/not-written.html %}

### The "x" icon is not clearing the selection

{% include options/not-written.html %}

### Can users remove all of their selections in a multiple select at once?

{% include options/not-written.html %}
